# The cholesterol metabolite 27-hydroxycholesterol stimulates cell proliferation via ERβ in prostate cancer cells

**DOI:** 10.1186/s12935-017-0422-x

**Published:** 2017-05-11

**Authors:** Shaneabbas Raza, Megan Meyer, Casey Goodyear, Kimberly D. P. Hammer, Bin Guo, Othman Ghribi

**Affiliations:** 1 Department of Biomedical Sciences, University of North Dakota School of Medicine and Health Sciences, 501 North Columbia Road, Grand Forks, ND 58202 USA; 2 Department of Veteran Affairs, Fargo VA Health Care System, Fargo, ND 58102 USA; 3 Department of Pharmaceutical Sciences, North Dakota State University, Fargo, ND 58108 USA

**Keywords:** 27-Hydroxycholesterol, Estrogen receptor, Hypercholesterolemia, Proliferation, Prostate cancer

## Abstract

**Background:**

For every six men, one will be diagnosed with prostate cancer (PCa) in their lifetime. Estrogen receptors (ERs) are known to play a role in prostate carcinogenesis. However, it is unclear whether the estrogenic effects are mediated by estrogen receptor α (ERα) or estrogen receptor β (ERβ). Although it is speculated that ERα is associated with harmful effects on PCa, the role of ERβ in PCa is still ill-defined. The cholesterol oxidized metabolite 27-hydroxycholesterol (27-OHC) has been found to bind to ERs and act as a selective ER modulator (SERM). Increased 27-OHC levels are found in individuals with hypercholesterolemia, a condition that is suggested to be a risk factor for PCa.

**Methods:**

In the present study, we determined the extent to which 27-OHC causes deleterious effects in the non-tumorigenic RWPE-1, the low tumorigenic LNCaP, and the highly tumorigenic PC3 prostate cancer cells. We conducted cell metabolic activity and proliferation assays using MTS and CyQUANT dyes, protein expression analyses via immunoblots and gene expression analyses via RT-PCR. Additionally, immunocytochemistry and invasion assays were performed to analyze intracellular protein distribution and quantify transepithelial cell motility.

**Results:**

We found that incubation of LNCaP and PC3 cells with 27-OHC significantly increased cell proliferation. We also demonstrate that the ER inhibitor ICI 182,780 (fulvestrant) significantly reduced 27-OH-induced cell proliferation, indicating the involvement of ERs in proliferation. Interestingly, ERβ levels, and to a lesser extent ERα, were significantly increased following incubation of PCa cells with 27-OHC. Furthermore, in the presence of the ERβ specific inhibitor, PHTPP, 27-OHC-induced proliferation is attenuated.

**Conclusions:**

Altogether, our results show for the first time that 27-OHC, through ER activation, triggers deleterious effect in prostate cancer cell lines. We propose that dysregulated levels of 27-OHC may trigger or exacerbate prostate cancer via acting on ERβ.

## Background

Prostate cancer (PCa) is the second leading cause of death among men in the United States [1]. The causes for PCa appear to be multifactorial, however it is well established that the incidence of PCa increases with age [1, 2]. Several risk factors are associated with PCa including aging [2], obesity [3], hormonal imbalance [3], oxidative stress [3, 4] and hypercholesterolemia [5, 6]. Estrogen receptor (ER) signaling has been implicated in PCa; the isoform ERβ, and to a lesser extent ERα, is expressed in prostate epithelial and stromal cells [7, 8]. ERα is considered pro-proliferative [9] and ERβ anti-proliferative in the context of PCa [10–12]. However ERβ agonists have not shown clinical promise to combat PCa [13] and there is a gap in knowledge elucidating the role of ERβ in PCa.

27-Hydroxycholesterol (27-OHC) is the most abundant cholesterol metabolite in the periphery [14, 15]. Also, 27-OHC in plasma increases with age, especially in men [16]. Men also have higher basal levels of 27-OHC in the plasma than women [16]. Moreover, while patients with hypercholesterolemia are at risk for developing PCa [6], they also have increased 27-OHC levels in the blood [17–19]. Understanding the role of 27-OHC in the context of PCa is critical and may reveal the underlying mechanisms responsible for PCa tumor initiation and progression. 27-OHC is a selective estrogen receptor modulator (SERM) that has been identified to bind to ER and modulate its activity [20, 21]. 27-OHC levels are higher among those with hypercholesterolemia [17–19] and older men [16], both of which are at high risk of developing PCa. Also, 27-OHC-induced ER activation has been shown to promote ER+ breast cancer growth and progression [18, 22–24]. Given that 27-OHC, at high levels, is associated with risk factors for PCa (i.e., hypercholesterolemia and aging) and that 27-OHC modulates ER signaling, a pathway that plays a role in PCa development and progression, it is essential to study the role of 27-OHC in the context of PCa. Further understanding of the role of 27-OHC in PCa may innovate alternative therapeutic avenues to those that are currently on the market.

We have previously shown that 27-OHC stimulates cell proliferation and inhibits docetaxel induced apoptosis in non-tumorigenic prostate epithelial cells [25]. In this study, we determined the extent to which 27-OHC is deleterious to PCa cells involving the activation of ERs.

## Methods

### Reagents

27-Hydroxycholesterol was purchased from Santa Cruz Biotechnologies (Dallas, TX), docetaxel, 4-[2-phenyl-5,7-bis(trifluoromethyl) pyrazolo[1,5-a]pyrimidin-3-yl]phenol (PHTPP) and fulvestrant from Cayman Chemicals (Ann Arbor, MI) and β-estradiol from Sigma-Aldrich (St. Louis, MO). All cell culture reagents, with the exception of fetal bovine serum (FBS) (Atlanta Biologicals; Flowery Branch, GA) were from Invitrogen (Carlsbad, CA). Human RWPE-1, LNCaP and PC3 cells were purchased from ATCC (Manassas, VA).

### Cell culture

Non-tumorigenic human prostate epithelial RWPE-1 cells were maintained in Keratinocyte serum free medium (Invitrogen; Carlsbad, CA) supplemented with 0.05 mg/ml BPE and 5 ng/ml EGF. Metastatic LNCaP cells were maintained in RPMI 1640 medium and highly metastatic PC3 cells were maintained in F-12K medium. LNCaP and PC3 cells were supplemented with 10% FBS. All cells were supplemented with 100 U/ml penicillin, 100 μg/ml streptomycin 0.25 μg/ml amphotericin (Sigma; St. Louis, MO) and cultured at 5% CO_2_ and 37 °C. Stock solutions of 27-OHC were prepared in 100% ethanol and stored at −80 °C. 27-OHC stock solution was dissolved in appropriate volumes of media to prepare the working solutions of 1 μM, a concentration that we showed to cause proliferation in prostate epithelial cells [25]. Stock solutions of β-estradiol were dissolved in 100% ethanol and stored at −80 °C. Stock solutions were diluted to prepare working solutions of 2 nM. Stock solutions of PHTPP and fulvestrant were also dissolved in 100% ethanol, stored at −20 °C, and diluted prior to treatment to prepare working solutions of 10 μM. Concentrations of solvent in treatments were less than 0.1%.

### Cell proliferation assay

Proliferation assays were conducted on black 96 well plates using CyQUANT Direct Cell Proliferation Assay (Invitrogen; Carlsbad, CA), which quantifies cell number using DNA content and membrane integrity. Cells seeded at 50–60% confluence were treated and incubated for 48 h. Cells were then stained as per the manufacturer’s protocol and read using Spectra MAX GEMINI EM (Molecular Devices; Sunnyvale, CA).

### Metabolic activity assay (MTS assay)

Cell metabolic activity was quantified by measurement of the reduction of MTS to formazan product using CellTiter 96^®^ AQ_ueous_ One Solution Cell Proliferation Assay (Promega; Madison, WI) according to the manufacturer’s protocol. The assay of the formation of formazan was performed by measuring absorbance change using a microplate reader (Molecular Devices; Sunnyvale, CA) 48 h after treatments.

### Western blot analysis

Treated cells were washed with PBS, trypsinized, and centrifuged at 5000*g*. The pellets were washed with PBS and homogenized in M-PER tissue protein extraction reagent (Thermo Scientific; Waltham, MA) supplemented with protease and phosphatase inhibitors. Denatured proteins (5 µg) were separated in 10% SDS-PAGE gels, transferred to a PVDF membrane (Millipore; Billerica, MA) and incubated with antibodies to ERα (1:1000, Santa Cruz; Dallas,TX) and ERβ (1:1000, Millipore, Billerica, MA). β-actin was used as a gel loading control. The blots were developed with enhanced chemiluminescence (ECL Clarity kit, Bio-Rad). Bands were visualized on a polyvinylidene difluoride membrane and analyzed by LabWorks 4.5 software on a UVP Bioimaging System. Quantification of results was performed by densitometry and the results analyzed as total integrated densitometric values (arbitrary units).

### Invasion assay

Invasion assays were conducted using QCM 96-well Cell Invasion Assay kit (Millipore; Billerica, MA). The various treatments were added to the wells of the feeder tray and at least 1 × 10^4^ cells/well re-suspended in serum free medium were added to the invasion chambers. Cells were incubated with treatments for 24 h then stained as per manufacturer’s protocol and transferred to a black 96 well plate to read fluorescence using Spectra MAX GEMINI EM (Molecular Devices; Sunnyvale, CA).

### Real-time polymerase chain reaction (RT-PCR)

After treatments, cells were lysed according to QuickGene Mini80 protocol and kit (Autogen). RNA sample were quantitated by spectrophotometry and subsequently, 1 µg total RNA was used as template to synthesize cDNA with the High Capacity cDNA Reverse Transcription Kit (Applied Biosystems; Foster City, CA). We selected genes involved in pathways related to oxysterols, such as 27-OHC, including cholesterol metabolism [26], liver X receptor (LXR) [27] and sonic hedgehog (Shh) [28] signaling. We also selected genes involved in metastasis [29–31], oxysterol binding [32] and tumor suppression [33]. The screened genes are included in Table 1. Primers for all assays were designed using Primer Express 3.0 (Applied Biosystems; Foster City, CA). Melting curve analysis was performed to ensure single-product amplification for all primer pairs. Real time PCR was performed on the ABI 7900HT Fast Real Time PCR System (Applied Biosystems; Foster City, CA) using the panel of genes of interest. Data analysis was performed using Sequence Detection System software from Applied Biosystems, version 2.4. The experimental Ct (cycle threshold) was calibrated against the endogenous control products alpha-ACTIN (ACTN1) and beta-2-microglobulin (B2M). Samples were analyzed for relative gene expression by the DDCt method [34].

**Table 1:** Selected genes analyzed with their corresponding pathways

Cholesterol metabolism (C)			Nuclear receptor signaling (N)			Shh signaling (S)			Metastasis (M)			Oxysterol binding (O)		Tumor suppression (T)		
CYP27A1	CYP7B1	HMGCR	ABCA1	EZH2	FOXA1	GLI1	GLI2	GLI3	CBX1	CBX5	CTGF	INSIG2	OSBP	MDM2	TAF3	TNF
NFKB1	SRD5A2	SREBF1	NR1H2	NR1H3	RXRA	PTCH1	PTCH2	SHH	FN1	IGF-1	IGFBP3			TP53		
			RXRB	SIRT1	TFF1	SMO			MTA1	MTA3	SNAI1					
			TMPRSS2						SNAI2	SPARC	VEGFA					

### Immunocytochemistry (ICC)

Coverslip seeded cells were rinsed with PBS and fixed in cold acetone, blocked with 10% normal goat serum and incubated overnight at 4 °C with human anti-ERβ2 monoclonal antibody (Biorad; Hercules, CA). ERβ2 was conjugated to Alexa Fluor 488. All coverslips were washed and mounted with Vectashield containing DAPI. Slides were visualized using DMI 6000 (Leica Microsystems; Buffalo Grove, IL).

### Statistical analysis

The significance of differences were assessed by unpaired *t* test and One Way Analysis of Variance (One Way ANOVA) followed by Tukey’s post hoc test. Statistical analysis was performed with GraphPad Prism software 4.01. Quantitative data for experimental analysis are presented as mean values ± SEM with unit value assigned to control and the magnitude of differences among the samples being expressed relative to the unit value of control.

## Results

### The cholesterol metabolite 27-OHC increases cell proliferation in PCa cells

**Fig. 1:**
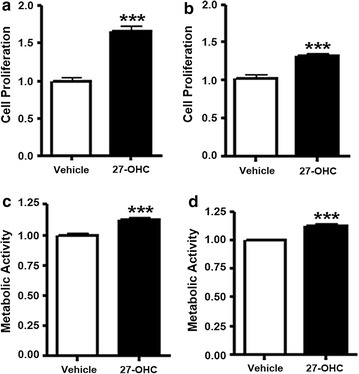
27-OHC induces cell proliferation in PCa cells. Cell proliferation assay in LNCaP (**a**) and PC3 (**b**) cells demonstrates a significant increase in proliferation in the presence of 27-OHC. MTS assay shows a significant increase in cell metabolic activity in the presence of 27-OHC in LNCaP (**c**) and PC3 (**d**) cells. Cells were treated with 1 µM 27-OHC. Readings were recorded 48 h after treatment with 27-OHC. Data is expressed as mean ± SEM. ***p < 0.001 versus controls

We have previously shown that 27-OHC stimulates cell proliferation in non-tumorigenic RWPE-1 cells [25]. However, the effects of 27-OHC on proliferation in PCa cells was not determined. Here we show that 27-OHC stimulates cell proliferation in PCa cells, LNCaP and PC3. Upon 27-OHC treatment, cell proliferation was increased by ~60% in LNCaP and ~30% in PC3 compared to their respective controls (Fig. 1a, b). To confirm our results, we performed MTS assay which measures mitochondrial activity of the cells. We found that 27-OHC also significantly increases metabolic activity of the both cells (Fig. 1c, d). These results suggest that 27-OHC induces cell proliferation in PCa cells.

### 27-OHC stimulates cell proliferation via ER

**Fig. 2:**
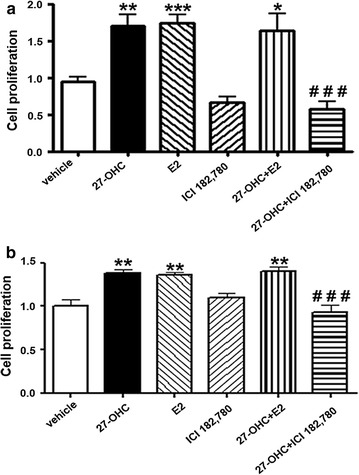
27-OHC stimulates cell proliferation via ER. Cell proliferation assay in LNCaP (**a**) and PC3 (**b**) cells demonstrates an attenuation of 27-OHC-induced cell proliferation with the ER inhibitor ICI 182,780 (fulvestrant). Cells were treated with 1 µM 27-OHC, 2 nM of E2 and 10 µM ICI 182,780. Readings were recorded 48 h after treatment with 27-OHC. Data is expressed as mean ± SEM. **p < 0.01; ***p < 0.001 versus controls, ^###^p < 0.001 versus 27-OHC only treatment

Since 27-OHC is a ligand of ER [21] and that 27-OHC-induced ER modulation leads to increased cell proliferation in the breast cancer cells [18, 22–24], we assessed the importance of ER in 27-OHC-induced cell proliferation in PCa cells. We have previously shown that 27-OHC induced cell proliferation in non-tumorigenic prostate epithelial cells was ER dependent [25]. Here, we show that the ER specific inhibitor ICI 182,780 (fulvestrant) [35] mitigated 27-OHC induced cell proliferation to basal levels in LNCaP and PC3 cells (Fig. 2a, b). Also, we found that upon concomitant treatment of 27-OHC and estradiol (E2), the natural agonist of ER [36], there was no additive effect in cell proliferation in both cells (Fig. 2a, b). These results suggest that ER activation is necessary for 27-OHC induced cell proliferation.

### 27-OHC selectively up-regulates ERβ expression

**Fig. 3:**
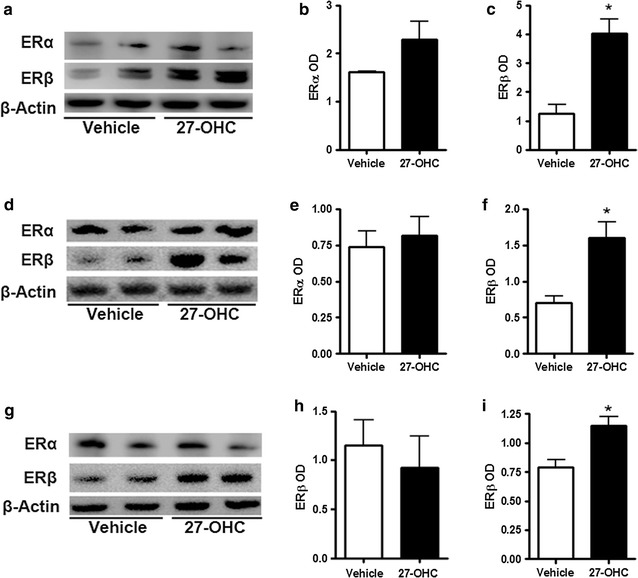
27-OHC upregulates ERβ expression. Representative western blots (**a**) and densitometric analysis showing no significant change in ERα expression in RWPE-1 (**b**) and a significant increase in ERβ expression (**c**) in RWPE-1 cells. Representative western blots (**d**) and densitometric analysis showing no significant change in ERα expression (**e**) and a significant increase in ERβ expression in LNCaP cells. Representative western blots (**g**) and densitometric analysis showing no significant change in ERα expression (**h**) and a significant increase in ERβ expression in PC3 cells (**i**). Data is expressed as mean ± SEM. *p < 0.05 versus controls

Given that 27-OHC stimulates cell proliferation in non-tumorigenic [25] as well as PCa cells (Fig. 1a, b) and that 27-OHC is a ligand of ER [21, 37], we determined the extent to which 27-OHC regulates ER protein levels. We found that while it has no significant effects on ERα levels, 27-OHC significantly upregulated ERβ levels in the non-tumorigenic RWPE-1 (Fig. 3a–c) and PCa cells (Fig. 3d–i). When compared to vehicle treated, 27-OHC treated cells exhibit an increase in ERβ levels by ~250% in RWPE-1 (Fig. 3c), ~100% in LNCaP (Fig. 3f), and ~50% in PC3 (Fig. 3i). This data suggests a potential involvement of ERβ in 27-OHC-induced cell proliferation.

### 27-OHC induces cell proliferation via ERβ

**Fig. 4:**
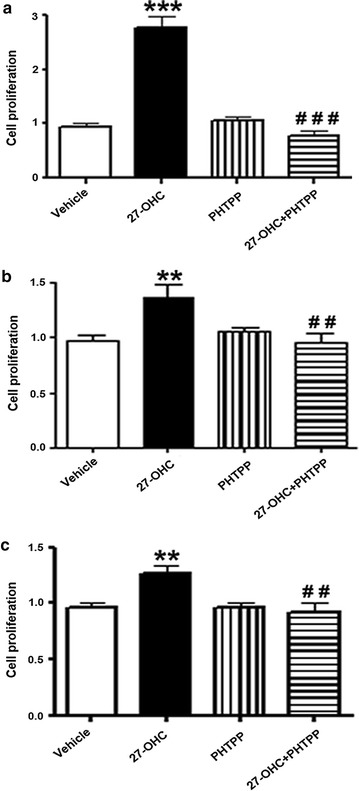
27-OHC induces cell proliferation via ERβ. Cell proliferation assay in RWPE-1 (**a**), LNCaP (**b**) and PC3 (**c**) cells demonstrates attenuation of 27-OHC-induced cell proliferation with PHTPP treatment. Cells were treated with 1 µM 27-OHC and 10 µM PHTPP. Readings were recorded 48 h after treatments. Data is expressed as mean ± SEM. **p < 0.01; ***p < 0.001 versus controls, ^##^p < 0.01; ^###^p < 0.001 versus 27-OHC only treatment

To determine if 27-OHC-induced cell proliferation is due to ERβ specific activation, we utilized an ERβ specific antagonist, PHTPP [38]. Upon treatment of the non-tumorigenic and PCa cells with 27-OHC and PHTPP, PHTPP attenuated 27-OHC-induced cell proliferation to basal levels in all cells (Fig. 4a–c). This data suggests that ERβ activation is essential for 27-OHC-induced cell proliferation.

### 27-OHC reduces cell invasion in PCa but not in non-tumorigenic cells

**Fig. 5:**
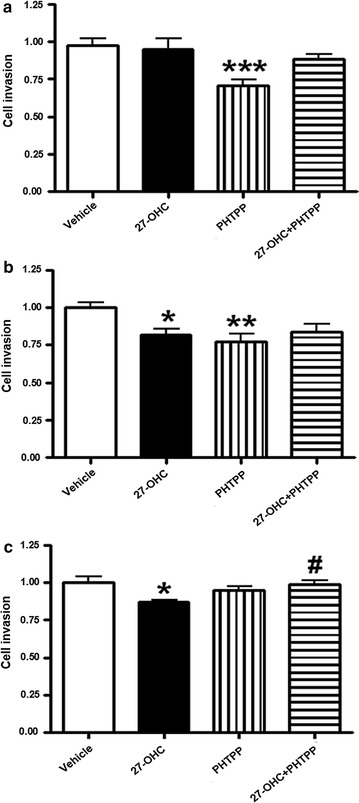
27-OHC reduces ECM invasion in PCa cells but not in non-tumorigenic cells. Cell invasion assay demonstrates that while there was no change in invasion in RWPE-1 cells treated with 27-OHC (**a**), a significant decrease in cell invasion occurred in LNCaP (**b**) and PC3 (**c**) cells treated with 27-OHC. Cells were treated with 1 µM 27-OHC and 10 µM PHTPP. Readings were recorded 48 h after treatment with 27-OHC. Data is expressed as mean ± SEM. *p < 0.05, **p < 0.01, ***p < 0.001 versus controls, ^#^p < 0.05 versus 27-OHC only treatment

Cell invasion is a key process by which cancerous cells further tumor progression and metastasize to distant tissues and organs. Metastatic cells invade healthy tissue by penetrating through the extracellular matrix (ECM) of healthy cells [39]. To investigate the effect of 27-OHC on cell invasion, we treated cells with 27-OHC and determined the change in cell invasion across the ECM. We found that upon 27-OHC treatment, cell invasion did not significantly change in the RWPE-1 cells (Fig. 5a) but significantly decreased in LNCaP and PC3 cells (Fig. 5b, c). Also, upon PHTPP treatment only, cell invasion significantly decreased in non-tumorigenic RWPE-1 and LNCaP cells but not in PC3 cells (Fig. 5a–c). Interestingly, PHTPP rescued 27-OHC-induced decrease in cell invasion of PC3 cells (Fig. 5c). This data suggests that 27-OHC has no effect on normal prostate but inhibits cell invasion in PCa cells.

### 27-OHC differentially regulates gene expression in non-tumorigenic and PCa cells

We determined the extent to which 27-OHC related gene expression is regulated in normal prostate epithelial RWPE-1 cells and PCa cells (Table 2). We found that in non-tumorigenic RWPE-1 cells, the expression levels of TFF1 (PS2) and TMPRSS2 were significantly upregulated by 27-OHC treatment. In LNCaP cells, CTGF, IGFBP-3, INSIG2, NR1H2 and RXRB expressions were significantly upregulated while SREBF-1 and TMPRSS2 expression were significantly down regulated. In PC3 cells, CBX1, CBX5, CYP27A1, CTGF, FOXA1, GLI2, GLI3, MDM2, MTA3, OSBP, PTCH1, RXRB and SIRT1 expression were significantly upregulated and only SPARC expression was significantly downregulated (Table 2). The sequences for primers of the significantly regulated genes are presented in Table 3. This data emphasizes the inherent differences in the cells and the potential genes regulated by 27-OHC in the different prostate cell lines.

**Table 2:** Significantly regulated genes in absolute fold change

Genes	RWPE-1	LNCaP	PC3
CBX1(M)	0.9956	1.0503	1.2222*
CBX5(M)	1.1252	1.0153	1.2895**
CTGF(M)	0.8859	3.5324*	1.4869**
CYP27A1(C)	0.5065	N/E	1.2263**
FOXA1(N)	1.1357	1.1356	1.1534**
GLI2(S)	0.9029	0.8980	1.3012***
GLI3(S)	0.9287	0.9672	1.1714*
IGFBP3(M)	0.9773	2.3619*	1.1511
INSIG2(O)	0.9938	1.1923*	1.1851
MDM2(T)	1.0007	1.0855	1.2051*
MTA3(M)	1.0713	1.1684	1.2135**
NR1H2(N)	0.9437	1.1690*	1.1723
OSBP(O)	1.1333	1.1635	1.2088*
PTCH1(S)	0.9376	1.3609	1.3304*
RXRB(N)	1.1306	1.2035*	1.2004**
SREBF1(C)	0.6093	0.6811*	1.1461
SIRT1(N)	0.9817	1.1805	1.2671*
SPARC(M)	0.9115	1.0014	0.8155***
TFF1(N)	1.8358*	1.3250	1.0190
TMPRSS2(N)	1.4082*	0.8474*	1.1262

*N/E* not expressed

* p < 0.05, ** p < 0.01, *** p < 0.001 versus controls

**Table 3:** Primers used in determining mRNA expression of significantly regulated genes

Gene	Sequence
CBX1-f	TGAGCAGCGTCACCCTTTACAC
CBX1-r	CCACTTTGCCCTTTACCACTCG
CBX5-f	TGGCACAATCTTGGCTTACTGT
CBX5-r	ATGGTGGCACACACCTGTAGTC
CTGF-f	AGGATGTGCATTCTCCAGCCATC
CTGF-r	TGTCAGAGCTGAGTCTGCTGTTC
CYP27A1-f	CAGCTGCGCTTCTTCTTTCAGC
CYP27A1-r	TGGCCTTGTAAAGCACCTGTAAC
FOXA1-f	TCCTCAGGAATTGCCCTCAAGAAC
FOXA1-r	ATGACATGACCATGGCACTCTGC
GLI2-f	TGTCTGAGTGACACCAACCAGAAC
GLI2-r	TGTGAATGGCGACAGGGTTGAC
GLI3-f	CCTCCAGCACCACTTCTAATGAGG
GLI3-r	TCTGTGGCTGCATAGTGATTGCG
IGFBP3-f	TCCAAGCGGGAGACAGAATATGG
IGFBP3-r	AGGAACTTCAGGTGATTCAGTGTG
INSIG2-f	CATGCCAGTGCTAAAGTGGATTTC
INSIG2-r	TGGATAGTGCAGCCAGTGTGAG
MDM2-f	TCCTCTCAAGCTCCGTGTTTG
MDM2-r	TCATGATGTGGTCAGGGTAGATG
MTA3-f	GCAGCAGAAGCTGAGAGTAAACTG
MTA3-r	TGGTTGGGATTTGGTTTGCTGTAG
NR1H2-f	GCATCCACTATCGAGATCATGCTG
NR1H2-r	GAAGGTGATACACTCTGTCTCGTG
OSBP-f	CTATGAAAGCCACAGAGGATGGC
OSBP-r	GTCCTTCTTCCGCTCAAACCAC
PTCH1-f	TCACCGTTCACGTTGCTTTGGC
PTCH1-r	AAACATGTGCTCCAGGGCAAGC
RXRB-f	TGCTGTGGAACAGAAGAGTGACC
RXRB-r	ATGTTAGTCACAGGGTCATTTGGG
SIRT1-f	ACAGGTTGCGGGAATCCAAAGG
SIRT1-r	CCTAGGACATCGAGGAACTACCTG
SPARC-f	TGGCGAGTTTGAGAAGGTGTGC
SPARC-r	TGGCAAAGAAGTGGCAGGAAGAG
SRD5A2-f	ACATACGGTTTAGCTTGGGTGTC
SRD5A2-r	TTTCTCCAGGCTTCCTGAGCTG
SREBF1-f	GCCATGGATTGCACTTTCGAAGAC
SREBF1-r	GGTCAAATAGGCCAGGGAAGTCAC
TAF3-f	GCCATCGGTACTCTGAGCTCTATG
TAF3-r	TGACGGAATTTGGTGTGGGAAGG
TFF1-f	GCCCCCCGTGAAAGACA
TFF1-r	CGTCGAAACAGCAGCCCTTA
TMPRSS2-f	TGTGGTCCCTTCCAATGCTGTG
TMPRSS2-r	TGCTCATGGTTATGGCACTTGGC
TNF-f	CCAGGCAGTCAGATCATCTTCTCG
TNF-r	ATCTCTCAGCTCCACGCCATTG
TP53-f	AGTGGAAGGAAATTTGCGTGTGG
TP53-r	TGGTACAGTCAGAGCCAACCTC
VEGFA-f	AGGGCAGAATCATCACGAAGTGG
VEGFA-r	AGGGTCTCGATTGGATGGCAGTAG
ACTINalpha-f	CAGGACCGTGTGGAGCAGATTG
ACTINalpha-r	CAGATTGTCCCACTGGTCACAG
B2M-f	TGAGTATGCCTGCCGTGTGAAC
B2M-r	TGCTGCTTACATGTCTCGATCCC

### 27-OHC regulates ERβ2 expression and cellular localization

**Fig. 6:**
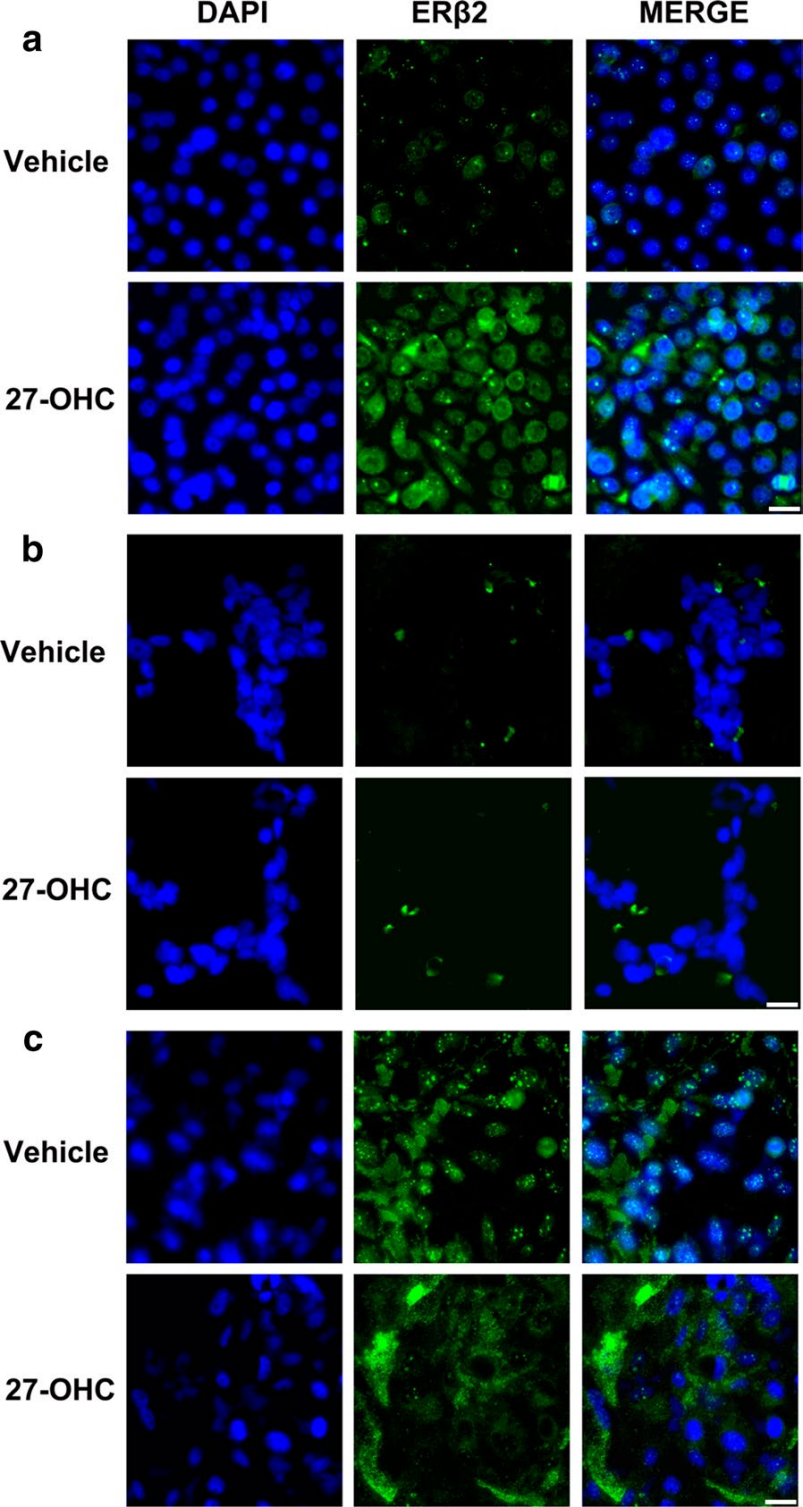
27-OHC differentially regulates ERβ2. Representative fluorescence microscopy images of prostate cells depicting ERβ2 (*green*) expression and localization and the nucleus (*blue*). **a** RWPE-1 cells treated with 27-OHC showed increased intensity of ERβ2. **b** LNCaP cells treated with 27-OHC depicted no change in ERβ2 staining intensity and **c** PC3 cells treated with 27-OHC demonstrated no change in overall ERβ2 staining intensity, but a decrease in nuclear ERβ2. All cells were treated with 1 µM 27-OHC for 24 h. *Bar* 50 µm

ERβ is categorized into several isoforms including ERβ1, ERβ2, ERβ4 and ERβ5 which are expressed in the prostate gland [40]. ERβ1 is the only functional isoform with a ligand binding domain while the activity of the other isoforms may depend on ERβ1 expression and isoform ratios [40]. The ERβ1 isoform is the most studied isoform which is known to have a protective role in prostate cancer while ERβ2 is considered deleterious [41, 42] and known to correlate with poor prognosis [43]. To test whether 27-OHC regulates ERβ2 expression in prostate cells, we treated cells with 27-OHC and stained for ERβ2. We found that while ERβ2 is expressed in a punctated fashion in the nucleus and the cytoplasm in RWPE-1 cells, 27-OHC appeared to increase overall ERβ2 expression (Fig. 6a). In LNCaP cells, we saw no changes in ERβ2 staining intensity (Fig. 6b.) and in PC3 cells we found that 27-OHC appeared to have no overall effect on ERβ2 expression, however interestingly, stained ERβ2 punctates in the nucleus decreased when treated with 27-OHC. This data suggests that 27-OHC alters the ERβ2 expression and cellular localization depending on the prostate cell line.

## Discussion

This study investigated the role of 27-OHC in PCa cell models. We demonstrate that 27-OHC induces cell proliferation in PCa cells through ER. We further show that 27-OHC regulates ERβ expression over ERα. Moreover, we demonstrate that 27-OHC-induced cell proliferation is dependent on ERβ activation. We also show that 27-OHC reduces ECM cell invasion in PCa cells but not in non-tumorigenic cells. Additionally, we found that among pre-selected genes, several genes that are related to oxysterol biology and PCa were significantly upregulated/downregulated following 27-OHC treatment in non-tumorigenic and PCa cells. These include TFF1 and TMPRSS2 in non-tumorigenic cells and CTGF and RXRB in PCa cells. We also demonstrated that 27-OHC may regulate ERβ2 levels and its cellular localization. Thus, our data show for the first time a potential link between 27-OHC and PCa pathogenesis by demonstrating the deleterious effect of 27-OHC in PCa cellular models.

Previously, we have reported that 27-OHC stimulates cell proliferation in non-tumorigenic prostate epithelial cells [25]. Subsequently, in this report, we demonstrate the effect of 27-OHC in PCa cells. Given that cell proliferation is associated with tumor growth, we measured cell proliferation upon 27-OHC treatment. We found that 27-OHC significantly increases cell proliferation in tumorigenic LNCaP and PC3 cells. Interestingly, the magnitude of 27-OHC-induced cell proliferation in LNCaP is higher than PC3. Also, the magnitude of 27-OHC-induced cell proliferation is higher in RWPE-1 than in LNCaP and PC3. The three epithelial cell lines have different morphologies, androgen receptor (AR) status, and depict different stages of PCa pathology. RWPE-1 are non-tumorigenic [44], LNCaP are androgen sensitive and with low tumorigenicity [45], and PC3 are androgen insensitive and highly tumorigenic [46]. Taking tumorigenicity of the cells into account, it appears that 27-OHC increases cell proliferation to a greater magnitude in prostate cells with low or non-tumorigenic phenotypes versus highly tumorigenic phenotype.

The role of estrogens and estrogen receptors in the context of PCa is currently being explored. Recently, estrogens have been associated with the development and progression of PCa [47]. Moreover, the discovery of 27-OHC as a SERM [21] adds another dimension to the growing phenomena of estrogenic actions as well as cholesterol metabolism role in PCas. Our previous [25] and current data strongly suggest that 27-OHC-induced cell proliferation in non-tumorigenic prostate cells and in PCa cells is ER dependent. When cells were treated concomitantly with 27-OHC and the ER inhibitor fulvestrant, proliferation was substantially attenuated. Given that 27-OHC is known to bind and activate ER [21, 23], our results suggest that activation of ER is required to ensue 27-OHC-induced cell proliferation in PCa cells. This observation suggests a potential link between 27-OHC, ER and PCa.

It is worth noting that being an ER modulator, 27-OHC can act as an agonist or antagonist depending on the target tissue. For example, 27-OHC activates ER in breast tissue [18, 22, 23] and inhibits the receptor in the vasculature [20]. We propose an additional novel concept that 27-OHC activates ER signaling in prostate cells and may thus play a pivotal role in PCa development and progression.

Furthermore, we demonstrate that 27-OHC-induced cell proliferation in non-tumorigenic and PCa cells is ERβ dependent. When cells are treated with the ERβ specific inhibitor PHTPP, the 27-OHC-induced cell proliferation is attenuated, suggesting the 27-OHC-induced ERβ activation as the underlying factor leading to cell proliferation.

Our results demonstrate that 27-OHC reduces cell invasion in PCa cells but not in non-tumorigenic cells. The finding that 27-OHC increases cell proliferation but reduces cell invasion is unexpected, however it is in accordance with the current environment of diagnosed PCa in which over 90% of PCa diagnosed are reported to be localized PCas [48, 49]. Additionally, it is important to note that cell invasion and proliferation are two different parameters in cancer. Moreover, androgen deprivation therapy (ADT) for patients with localized PCa does not improve survival or delay the use of secondary cancer therapy [50], suggesting a potential role of 27-OHC-induced estrogenic signaling in localized PCa.

Our study determined the expressions of genes regulated by 27-OHC in non-tumorigenic cells and PCa cells. In non-tumorigenic RWPE-1 cells, we found that 27-OHC treatment upregulated TFF1 (ps2) and TMPRSS2, downstream targets of ER [48] and AR [49], respectively. The upregulation of these target genes by 27-OHC demonstrates that this oxysterol activates target genes to both ER and AR activation. This corroborates with our earlier report that AR and ER are necessary to induce cell proliferation in the 27-OHC-treated RWPE-1 cells [25]. This observation also substantiates the idea that ER and AR activation simultaneously play a significant role in PCa tumor initiation. For instance, when Noble rats are concomitantly treated with estradiol and testosterone almost rats develop PCa tumors, while only 40% of them develop PCa when treated with testosterone alone [50, 51].

Unlike in RWPE-1 cells, we did not find upregulation of ps2 and TMPRSS2 genes by 27-OHC in PCa cells. This may be attributed to the fact that LNCaP has a mutated AR [52] and that PC3 has no AR [53]. In PCa cells, we found that the connective tissue growth actor(CTGF), which plays a vital role in tumorigenesis and wound healing processes [54] is upregulated by 27-OHC. Also, specific to LNCaP, we found a significant upregulation of insulin-like growth factor binding protein-3(IGFBP-3) which has been implicated in PCa tumors. IGFBP-3 is an anti-angiogenic and anti-metastatic protein that is upregulated and localized in the nucleus of PCa tumor cells [55].

Moreover, our results demonstrate that ERβ2 can be regulated by 27-OHC. In RWPE-1, the cells which obtained the most increase in cell proliferation upon 27-OHC treatment, ERβ2 expression increased. Surprisingly while no change was observed in LNCaP, PC3 demonstrated a reduction in nuclear ERβ2 expression, which corresponds to the decreased cell invasion upon 27-OHC treatment (Fig. 5c). This finding corroborates with the observation that nuclear ERβ2 abundance is associated with poor PCa prognosis and increased cell invasion [43].

Given the observations that 27-OHC induced AR transactivation and increased cell proliferation in an AR dependent manner in RWPE-1 cells, and 27-OHC does not directly bind to AR [25], RWPE-1 is the only cell line in this study with a wild type AR [44] and it is also the only cell line to show an increase in ERβ2 expression upon 27-OHC treatment. Furthermore, 27-OHC augmented cell proliferation at a greater magnitude in non-tumorigenic (RWPE-1) compared to low tumorigenic (LNCaP) and highly tumorigenic (PC3) PCa cells. Taken together, we hypothesize that 27-OHC binds and activates ERβ, inducing downstream AR-ERβ2 crosstalk signaling events leading to increased cell proliferation which may result into early stages of PCa. Further studies are warranted to test this hypothesis and determine the relationship between AR and ERβ2 in the presence of 27-OHC in the context of wildtype AR + prostate cancers.

Although there are variations between the both the PCa cells due to their difference in phenotypes, they have notable similarities. Both having mutated ARs; LNCaP having a mutated AR for increased androgen sensitivity and PC3 having the mutated AR for decreased androgen sensitivity, hence each cell line depicts a different stage of PCa. Also, 27-OHC increased proliferation, decreased cell invasion and increased expression of CTGF in both cell lines, CTGF is known to play anti-metastatic roles [56, 57]. These results establish a rationale and prelude to the potential role of 27-OHC in promoting tumor growth in localized prostate cancers.

## Conclusions

Altogether, our results demonstrate that 27-OHC induces an increase in cell proliferation in PCa cells. We also show for the first time that 27-OHC-induced cell proliferation is dependent on ER activation, specifically ERβ, in non-tumorigenic and PCa cells. Our study brings new insights into the potential role of 27-OHC-evoked effects on ERs in PCa development. Further studies delineating underlying mechanisms involved in 27-OHC induced ER-AR crosstalk in the context of PCa are warranted and may reveal novel therapeutic avenues to prevent, delay and/or attenuate PCa progression.

## Abbreviations

27-OHC: 27-hydroxycholesterol
ER: estrogen receptor
ERα: estrogen receptor α
ERβ: estrogen receptor β
PHTPP: 2-phenyl-5,7-bis(trifluoromethyl) pyrazolo[1,5-a]pyrimidin-3-yl]phenol
FBS: fetal bovine serum
PCa: prostate cancer
SERM: selective estrogen receptor modulator
